# Investigation of the Renal Protective Effect of Combined Dietary Polyphenols in Streptozotocin-Induced Diabetic Aged Rats

**DOI:** 10.3390/nu14142867

**Published:** 2022-07-13

**Authors:** Yassine Chtourou, Maram Morjen, Rahma Ammar, Rania Mhiri, Mohamed Jemaà, Ines ELBini-Dhouib, Hamadi Fetoui, Najet Srairi-Abid, Naziha Marrakchi, Jed Jebali

**Affiliations:** 1 Laboratory of Toxicology and Environmental Health (17ES06), Sciences Faculty of Sfax, University of Sfax, BP 1171, Sfax 3000, Tunisia; yacine_chtourou@yahoo.fr (Y.C.); ammar.rahma@outlook.fr (R.A.); rania8chaari@gmail.com (R.M.); fetoui_hamadi@yahoo.fr (H.F.); 2 The Higher Institute of Medical Technologies of Tunis (ISTMT), University of Tunis El Manar, 9 Street Doctor Zouheïr Safi, Tunis 1006, Tunisia; 3 Laboratory of Biomolecules, Venoms and Theranostic Applications, LR20IPT01, Pasteur Institute of Tunis, University of Tunis El Manar, Tunis 1002, Tunisia; maram.morjen@fst.utm.tn (M.M.); inesbini@yahoo.fr (I.E.-D.); najet.abidsrairi@pasteur.tn (N.S.-A.); naziha.marrakchi@pasteur.tn (N.M.); 4 Laboratory of Molecular Biotechnology of Eukaryotes, Sfax Biotechnology Centre, University of Sfax, BP 1177, Sfax 3038, Tunisia; jemaamohamed@gmail.com; 5 Medicine School of Tunis, University of Tunis El Manar, 15 Djebel Lakhdhar Street, La Rabta, Tunis 1007, Tunisia

**Keywords:** natural polyphenols, streptozotocin-induced diabetic aged rats (STZ), renal fibrosis, pharmacological properties

## Abstract

Natural polyphenols are widely reported to have a large range of pharmacological properties, especially antioxidant activities and free radical scavenging capacities. In this study, we investigate the effects of naringin, chlorogenic acid, and quercetin mixtures (NCQ) on renal fibrosis in streptozotocin (STZ)-induced diabetic aged rats and its underlying mechanisms for ten consecutive weeks. The oxidative defense system in the kidneys of treated rats was found to be improved. Several biomarkers were investigated including the blood urea nitrogen, creatinine, and uric acid. Moreover, antioxidant parameters were evaluated and we found that superoxide dismutase, catalase, glutathione peroxidase, Na^+^-K^+^-ATPase activities, the nitric oxide production, the protein carbonyl, the advanced oxidation protein products, lipid peroxidation, and reduced glutathione levels were all significantly balanced and close to control values. In addition, NCQ restored renal injuries and fibrosis as assessed by histological method and molecular biology investigation of the matrix metalloproteinase, the transforming growth factor-beta TGF-β, the tumor necrosis factor TNFα, and p53 expression. Our study proposes the NCQ combination as potential plant-derived bioactive compounds to prevent diabetic nephropathy.

## 1. Introduction

Diabetes mellitus is considered as one of the main causes of mortality and morbidity worldwide [1,2]. Diabetes prevalence is increasing yearly in a rapid way and prevalence estimations state 463 million people in 2019, rising to 578 million by 2030, and 700 million by 2045 globally [3]. In consequence, the World Health Organization (WHO) project diabetes-related death to double between 2005 and 2030. Indeed, in 2019, diabetes was the direct cause of 1.5 million deaths and 48% of all deaths due to diabetes occurred before the age of 70 years [4]. Diabetes mellitus is described as a chronic metabolic disease featuring hyperglycemia, insulin insufficiency, and excessive oxidative stress markers [5]. The oxidative stress is caused by a breakdown in the physiological balance between oxidative markers and anti-oxidative defense systems [6]. In fact, the oxidative stress leads to elevate intracellular levels of reactive oxygen species (ROS) that induces several cellular changes including damage to lipids, proteins, and DNA [7]. In diabetes, high blood glucose concentration increases the production of ROS and activates apoptosis in the β-cells of pancreas [8]. In fact, apoptosis and programmed necrosis play an important role in the progression of diabetic complications and provoke injuries in kidney [9,10,11]. Taken together, oxidative stress is directly linked to the pathophysiology of diabetic nephropathy [12,13]. The nephropathy is a severe micro-vascular complication of diabetes leading to kidney damage and a major cause of morbidity in the diabetic population. Indeed, about a third of the diabetics globally are diagnosed and classified with nephropathy [12,13,14]. The physiopathological mechanism of nephropathy is characterized by progressive structural alterations, including the thickening of glomerular and tubular basement membranes, glomerular lesions, elevated biochemical parameters such as serum creatinine, blood urea nitrogen, and tubule-interstitial fibrosis [15]. Renal fibrosis is a leading cause of kidney failure, and thus, controlling the progression to fibrosis remains a major challenge to prevent this consequence of diabetes [16,17]. To date, only a few therapeutic strategies exist, and have a moderated effect [18]. Moreover, relining only on diet restriction, widely prescribed as a solution, could have a negative effect, as diet provides numerous antioxidants, particularly vitamins and trace metals [15], and de facto, disruption of balanced diet affects the defense system and increases consequently the oxidative stress [19]. Polyphenols have important anti-oxidant properties as demonstrated by several studies and reports [20,21,22,23]. These properties presented them as major compounds in the prevention and treatment of chronic disease including cancer, cardiovascular disease (CVD), neurodegenerative disorders, diabetes, and renal failure [24,25,26,27]. Phenolic compounds are extensively distributed in plants and exhaustive studies demonstrated their benefits and curative potential, including antioxidant, anti-microbe, anti-neoplasic, and anti-inflammatory effects [28,29,30,31,32]. Phenolic compounds in foods such as naringin, quercetin, and chlorogenic acid has been found in a variety of herbs and fruits. These compounds were reported as having important antioxidant and anti-diabetic properties in several in vivo studies [33,34,35,36,37,38]. The aim of this study is to develop a new preventive and therapeutic possibility with polyphenols constituted by naringin, chlorogenic acid, and quercetin combination (NCQ) to prevent diabetic fibrosis using streptozotocin-induced diabetic aged rats.

## 2. Materials and Methods

### 2.1. Polyphenolic Compounds

Chlorogenic acid, quercetin, and naringin were obtained from Sigma Chemicals (St. Louis, France). In our laboratory we focus on polyphenol found in fruits, a regular food in Tunisian diet. Naringin, chlorogenic acid, and quercetin are de facto recurrently found in Tunisian plates. We performed a screen in PubMed with the key word naringin, chlorogenic acid, quercetin, STZ-induced diabetic rat and we found that in the majority of the papers, naringin was used at 25 to 50 mg/kg, quercetin at 25 to 100 mg/kg, and chlorogenic acid at 10 to 20 mg/kg. We decided then, based on this literature, to use the most cited concentration, meaning naringin (50 mg/kg), chlorogenic acid (10 mg/kg), and quercetin (50 mg/kg) [33,34,35,36,37,38].

### 2.2. Animals and Ethics

For our experimental protocol, 30 twelve-month-old Wistar rats (450 g ± 40 g) were obtained from Pasteur Institute of Tunis animal center and maintained under regulated light (12 h day/night cycle) in an air conditioned room (22 ± 2 °C) and humidity (50–60%) with free access to water and standard pelletized feeds (except in the fasting period). All experimental procedures were approved by the Tunisian Animal Ethics Committee (at the Faculty of Science of Sfax (FSS) (Ethical Approval No. 2015/14/E/FSS; 9 November 2015), in accordance with the guidelines for care and use of laboratory.

### 2.3. Induction of Diabetes

The diabetic rats models were established by single intraperitoneal injection of 45 mg/kg of streptozotocin (STZ) dissolved in freshly prepared citric acid-sodium citrate buffer (pH 4.2/4.5). Three days later, glycemia levels were measured in 18 h fasting rats by measuring blood glucose concentrations in blood samples obtained from the tails using a blood glucose meter (Accu-Chek blood glucose monitor, Roche, IN, USA). Rats having a glucose levels higher than 250 mg/dL were used as diabetics in the experiments.

### 2.4. Experimental Design

Animals were randomly divided into three groups: Control group (*n* = 10), STZ group (*n* = 10), and STZ treated with the mixture treatment group (naringin, chlorogenic acid, and quercetin) (*n* = 10). The mixture is dissolved in buffered saline and propylene glycol 25/75 (*v/v*) and administered for 10 weeks by oral gavage (6 days per week) without exceeding 1.0 mL/kg body weight. At the end of experimentation, rats were overnight fasted, and then sacrificed. Serum samples were isolated and kept frozen at −80 °C till biochemical analysis of kidney functions. In addition, kidneys were isolated, weighed, homogenized in saline, and kept frozen at −80 °C for the measurements of oxidative stress biomarkers and the histopathology study.

### 2.5. Determination of Serum Urea and Creatinine Concentration

Serum urea and creatinine were measured with spectrophotometric reagents commercially available (MAK006-1KT, MAK080-1KT, Sigma, Saint-Louis, MO, USA) and the absorbance was measured using Multiskan FC Microplate Photometer (Thermo Fisher Scientific, Waltham, MA, USA). Blood urea nitrogen (BUN) was calculated using the following formula: urea (mg/dL) × 0.467 = BUN (mg/dL).

### 2.6. Oxidative Stress Assessment

#### 2.6.1. Estimation of Nitric Oxide (NO) Production

Nitric oxide production was evaluated by measuring the nitrite, an oxidative product of NO. Nitrites were measured by Griess reaction by mixing 100 μL of supernatant with 100 μL of Griess reagent. The absorbance of each sample at 540 nm against a calibration curve with sodium nitrite standard was measured using a spectrophotometer (Thermo Fisher Scientific, Waltham, MA, USA).

#### 2.6.2. Lipid Peroxidation

MDA assay was analyzed using TMP reagent as the standard. Briefly, 100 μL of tissue extract was added to 50 μL of 8% sodium dodecyl sulphate (SDS) and incubated for 15 min at room temperature. A total of 380 μL of 20% acetic acid (pH 3.5) and 380 μL of 0.6% thiobarbituric acid (TBA) were added and then placed in a water bath for 1 h at 100 °C. The sample was then cooled at room temperature, and 1.25 mL of butanol: pyridine (15:1 *v*/*v*) mixture was added and centrifuged at 1500 rpm for 10 min. The absorbance was measured at 530 nm and the concentration was expressed in terms of nmoles MDA/g tissue.

#### 2.6.3. Carbonyl Protein Content (PCO)

Protein carbonyl (PCO) was determined using the method of Reznick and Packer [39]. Briefly, 10 mM of 2,4-dinitrophenylhydrazine (DNPH) in 2M HCl was added to 100 μL of tissue extract and incubated in obscurity for 1 h at room temperature. Samples were then mixed, and a solution of 25% trichloro acetic acid (TCA) was added. Sample was then washed twice with an ethanol ethyl acetate (1:1 *v*/*v*) mixture. The protein was precipitated and then solubilized in 6 M guanidine hydrochloride, 25 mM phosphate buffer pH (6.5). Insoluble materials were removed by centrifugation and the absorbance was measured at 370 nm with spectrophotometer. Carbonyl protein content was estimated based on the molar extinction coefficient of DNPH (ε = 2.2 × 104 cm^−1^ M^−1^) and expressed as nmoles per milligram of protein.

#### 2.6.4. Determination of AOPP Levels

Advanced oxidation protein product (AOPP) levels were determined according to the method of Kayali et al. [40]. The concentration of AOPP was measured using the extinction coefficient of 261 cm^−1^ mM^−1^ and the results were expressed as nmoles per milligram of protein.

#### 2.6.5. Catalase Activity

About 100 μL of the kidney extract was incubated with 100 μL pure ethanol for 40 min in ice. This step was primordial to release the full form of active enzyme. After 30 min, tubes were removed from the ice, maintained at room temperature. Then, 10 μL of Triton X-100 was added. To the reaction mixture containing 250 μL of phosphate buffer, 50 μL of the tissue extract and 300 μL of 0.066 M H_2_O_2_ in phosphate buffer were supplemented. After that, the absorbance was read at 240 nm for 30 s in a spectrophotometer. A molar absorptive of 43.6 M cm^−1^ was used to determine the catalase activity, one unit of which is equal to μmoles of hydrogen peroxide (H_2_O_2_) degraded per minute per mg of protein.

#### 2.6.6. Superoxide Dismutase SOD Activity

Superoxide dismutase SOD activity was calculated at room temperature using commercial assay kits (19160-1KT-F, Sigma, Saint-Louis, MO, USA). About 100 μL of the kidney extract was solubilized in 850 μL carbonate buffer (0.05 M, pH 10.2, 0.1 mM EDTA) and 50 μL of 30 mM epinephrine in 0.05% acetic acid. The mixture was incubated for 10 min. After incubation, the absorbance was measured at 480 nm using Multiskan FC Microplate Photometer (Thermo Fisher Scientific, Waltham, MA, USA). The amount of enzyme that results in 50% inhibition of epinephrine activity (SOD_50_) is defined as one unit.

#### 2.6.7. Estimation of Total GSH Levels

DTNB reagent was used to estimate reduced glutathione (GSH) level in tissue homogenates and the absorbance was read at 412 nm after 10 min. The amount of GSH in the sample was expressed in microgram per mL from a standard curve obtained and represented in µg per mg of protein.

#### 2.6.8. Determination of Glutathione Peroxidase Activity

Glutathione peroxidase activity (GPx) was measured according to Flohe and Gunzler [41]. The enzyme activity was expressed as nmol of GSH oxidized/min/mg protein.

#### 2.6.9. Na^+^-K^+^-ATPase Specific Activities

The ATPase activities were estimated after homogenizing the tissue extract in Tris–HCl buffer, pH 7.4. The supernatants were immediately used for ATPase determination following the method described by Kawamoto et al. [42]. Total ATPase activity was determined by Pi assay liberated from hydrolyzed adenosine triphosphate (ATP) forming a complex with molybdate. The ATPase activities were expressed as micromoles of Pi liberated per hour per mg of protein.

### 2.7. Protein Quantification

Protein concentration in kidney homogenates was measured by Bradford method using bovine serum albumin as standard.

### 2.8. Quantitative RT-qPCR

Expression of inducible TGF-β1, MMP-9, MMP-2, TNF-α, and P53 genes in the kidney tissues of experimental rats (*n* = 10/group) were measured using a reverse transcriptase qRT-PCR technique. Total RNA was extracted from renal tissue using the iScript™ RT-qPCR Sample Preparation Reagent (Bio-Rad, #1708899). RNA concentrations and purity were determined by measuring the absorbance A260/A280 ratios by using the Thermo Scientific NanoDrop spectrophotometer (Thermo Scientific™). The cDNA was synthesized by reverse transcription using the superscript reverse transcriptase (Invitrogen, France). About 3 μL cDNA was used as a template for PCR according to the recommended protocol using the 2× SYBR Permix Ex Taq™ (TaKaRa). The primer sets used for the gene’s amplifications are listed in Table 1. Data are reported as the fold change of control, following normalization against β-actin expression.

### 2.9. DNA Gel Electrophoresis

DNA fragmentation assay was carried out according to the method described by Sellins and Cohen [43]. The genomic DNA sample was isolated from renal tissue and was subjected to electrophoresis in a 1% agarose gel for 1 h at 100 V.

### 2.10. Histopathology Study

Kidneys were removed and fixed in a buffer solution of 10% formalin. Fixed tissues were processed routinely for paraffin embedding, and 6 µm sections were prepared, stained with hematoxylin eosin (H&E) and picrosirius red by using standard protocols. Stained areas were viewed and examined with an optical microscope (Leica MC170 HD, Leica microsystem, Wetzlar, Germany) connected with the Leica MC170 HD microscope camera for histological analysis. Fibrosis was assessed quantitatively by picrosirius red staining, which reacts specifically with collagen and does not stain other matrix proteins; morphometric analyses to estimate the percentage of surface area staining positively for collagen in each kidney was performed by Chtourou et al. [44]. Images of ten random fields were taken in each kidney. ImageJ v1.53K density software (National Institutes of Health, Bethesda, MD, USA) was used to calculate the percent area of each image that stained positive for Sirius Red, glomerular mesangial matrix expansion, and the average of 10 images per kidney was calculated.

### 2.11. Statistical Analysis

Results were expressed as mean ± standard deviation (mean ± SEM). All analyses were carried out with GraphPad Prism 4.02 for Windows (GraphPad Software, San Diego, CA). Significant differences between treatment effects were determined by one-way ANOVA, followed by Tukey’s post-hoc test for multiple comparisons with statistical significance of *p* < 0.05.

## 3. Results

### 3.1. NCQ Prevents the Alterations of Renal Biomarkers in STZ-Induced Diabetic Rats

**Figure 1:**
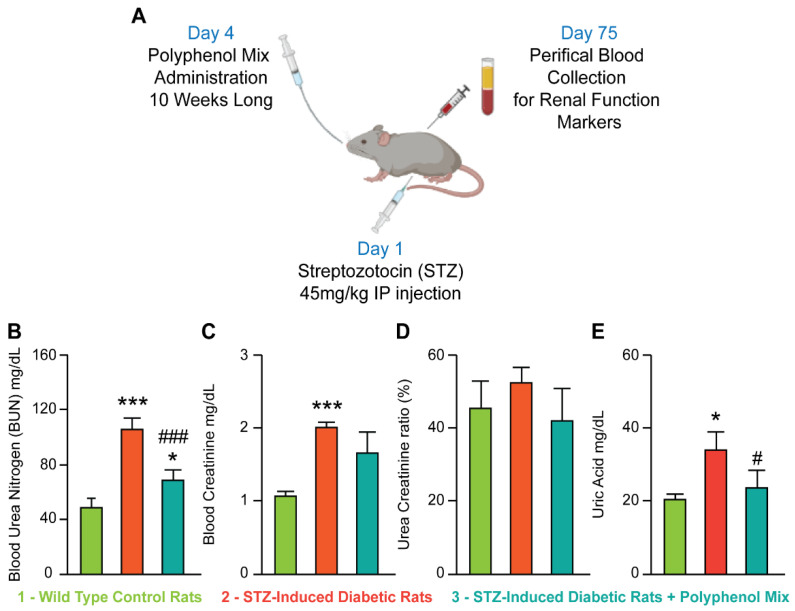
Diabetes induction model with STZ for exploring the renal protective effects of polyphenol mix. (**A**) Illustration of the study design. (**B**–**E**) Hematological parameters of urea, creatinine, urea creatinine ratio and uric acid of control, STZ-induced diabetic and treated rats with a combination of naringenic, chlorogenic acid, and quercetin (NCQ). Graphical synthesis of experimental STZ-induced diabetic rats was created with BioRender.com. Data are presented as means ± SEM. * *p* < 0.05, *** *p* < 0.001 indicate significant difference when the STZ-induced diabetic rats is compared to control group. # *p* < 0.05, ### *p* < 0.001 indicate statistical difference when NCQ-STZ treated rats are compared to STZ group.

To investigate the potential protective effect of a polyphenols mix on the renal tissue, we provoked renal failure in rats with streptozotocin (STZ). Indeed, at 45 mg/mL dose, STZ induces complete β-cell necrosis and de facto renal failure and diabetes [45]. We then treated our diabetic rats with a mix of polyphenols. Based on the literature, we prepared a combination of natural polyphenolic compounds, namely naringin, (50 mg/kg), chlorogenic acid (10 mg/kg), and quercetin (50 mg/kg) [46,47,48]. We baptized our polyphenol mix as NCQ for naringin, chlorogenic acid, and quercetin. After 10 weeks of treatment, we first evaluated several biochemical markers, with and without NCQ uptake, Figure 1A. As expected, STZ injection-induced renal failure and the levels of urea, creatinine, and uric acid in serum were significantly increased (*p* < 0.001) (Figure 1B–E) respectively, as compared to the levels in healthy control rats. However, NCQ treatment restored the control values and we observed a readjustment in BUN plus equilibrium of creatinine and uric acid in blood concentration (Figure 1B–E).

### 3.2. NCQ Ameliorates Renal Lipid Peroxidation, Nitric Oxide, Protein Oxidation and Glutathione Depletion in STZ-Induced Diabetic Aged Rats

**Figure 2:**
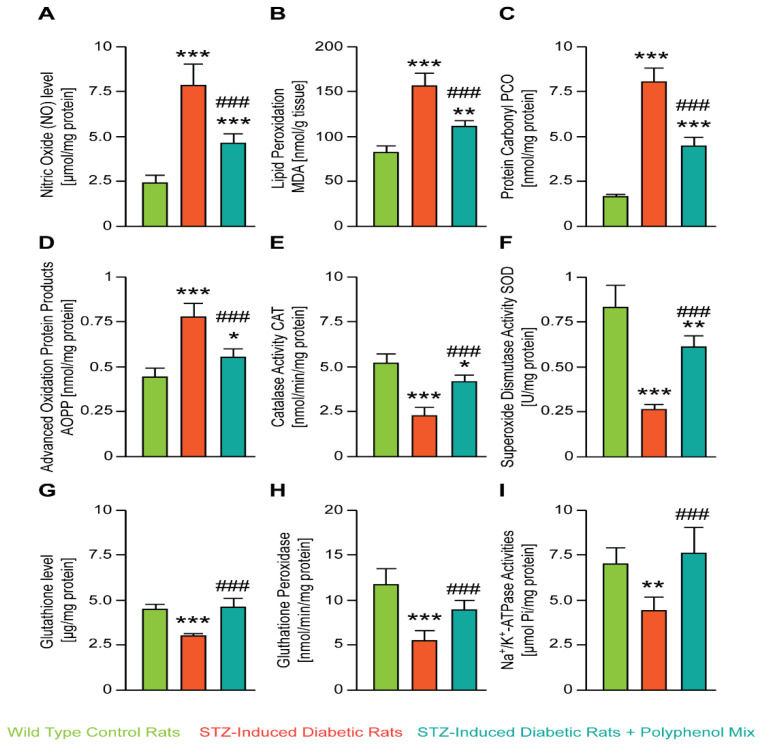
In vivo study and oxidative stress assessment. (**A**–**D**) Effect of polyphenol combination on kidney CAT, SOD, GPx, and Na^+^-K^+^-ATPase activities and GSH levels, in STZ-induced diabetic rats. (**E**–**I**) Effect of polyphenol mix on kidney CAT, SOD, GSH levels, GPx activity, and Na^+^-K^+^-ATPase activities in STZ-induced diabetic rats. Data are expressed as means ± SEM. *** *p* < 0.001, ** *p* < 0.01, * *p* < 0.05 when the STZ-induced diabetic rats is compared to control group; ### *p* < 0.001 when NCQ-STZ treated rats are compared to STZ group.

A next series of experiments explored whether the NCQ affected the oxidative stress induced by the STZ. We isolated renal tissue and measured respectively the nitric oxide (NO) level [µmol/mg protein] (Figure 2A), the lipid peroxidation MDA [nmol/g tissue] (Figure 2B), the protein carbonyl PCO [nmoles/mg protein] (Figure 2C), and advanced oxidation protein products AOPP [nmol/mg protein] (Figure 2D). STZ treatment significantly increased these oxidative stress biomarkers while NCQ co-treatment balanced the NO, PCO, AOPP, and MDA levels. As shown in Figure 2G, a significant decrease in GSH level (*** *p* < 0.001) in the kidney was observed in STZ group. The administration of NCG with STZ increased significantly this parameter when compared with STZ group.

In line with these data, STZ-induced diabetic rats showed also a significant decrease in the catalase activity CAT [nmol/min/mg protein] (Figure 2E), the superoxide dismutase activity SOD [U/mg protein] (Figure 2F), the activity of glutathione peroxidase GPx [nmol/min/mg protein] (Figure 2H), and also the Na^+^-K^+^-ATPase activities [μmol/Pi/mg protein] (Figure 2I). However, NCQ treatment elevated significantly the activity of these biomarkers and STZ-induced diabetic rats showed a near to normal values.

### 3.3. Investigation of NCQ on Genes Involved in Inflammation and Apoptosis Induced by STZ

**Figure 3:**
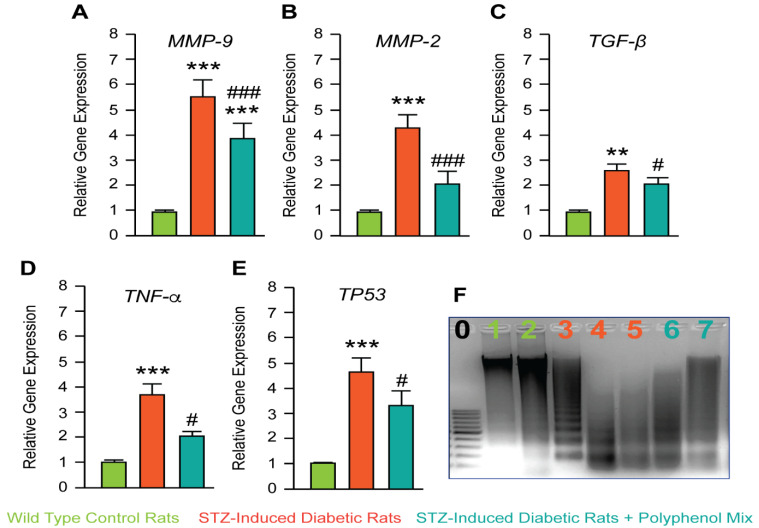
Studies investigating genes involved in inflammation and apoptosis. (**A**–**E**) Analysis of gene expression by quantitative RT-qPCR of MMP-9, MMP-2, TGF-β, TNFα, and P53 in aged rats treated for ten week with streptozotocin and polyphenol combination (NCQ). Data are reported as the fold change of control, following normalization with β-actin expression. (**F**) DNA fragmentation assay. Lane 1/2: DNA of control group. Lane 3/4/5 STZ group and 6/7 NCQ-STZ group. Data are expressed as means ± SEM. *** *p* < 0.001, ** *p* < 0.01, when the control group is compared to STZ-induced diabetic rats group; # *p* < 0.05, ### *p* < 0.001 when STZ-untreated rats are compared to NCQ-treated group.

To confirm the beneficial uptake of the NCQ mix, we decided to evaluate the matrix metalloproteinases MMP-9 and MMP-2 and the transforming growth factor-beta TGF-β expressions. At transcriptional levels, STZ-induced diabetic rats showed a significant increase of MMP-9, MMP-2, and TGF-β while treatment with NCQ partially, but significantly inhibited their transcription (Figure 3A–C). In line with these data, we studied the expression of two main genes involved in cell death-related fibrosis, namely the pro-inflammatory cytokines TNF-α and the guardian of the transcription factor p53. Both genes were over-transcripted in diabetic rats compared to control one; however, the NCQ treatment significantly reduced their expression (Figure 3D,E). To confirm the anti-fibrotic and thus anti-apoptotic effect of the NCQ treatment in rat’s kidney tissue, we performed a DNA fragmentation assay. To confirm the anti-fibrotic and thus anti-apoptotic effect of the NCQ treatment in rat’s kidney tissue, we performed a DNA fragmentation assay, and as expected, compared to diabetic rat tissue, treated rats with the phenolic mix showed a reduction in DNA degradation and de facto less fibrosis in kidney (Figure 3F).

### 3.4. Effect of NCQ in STZ-Induced Renal Histopathological Lesions

**Figure 4:**
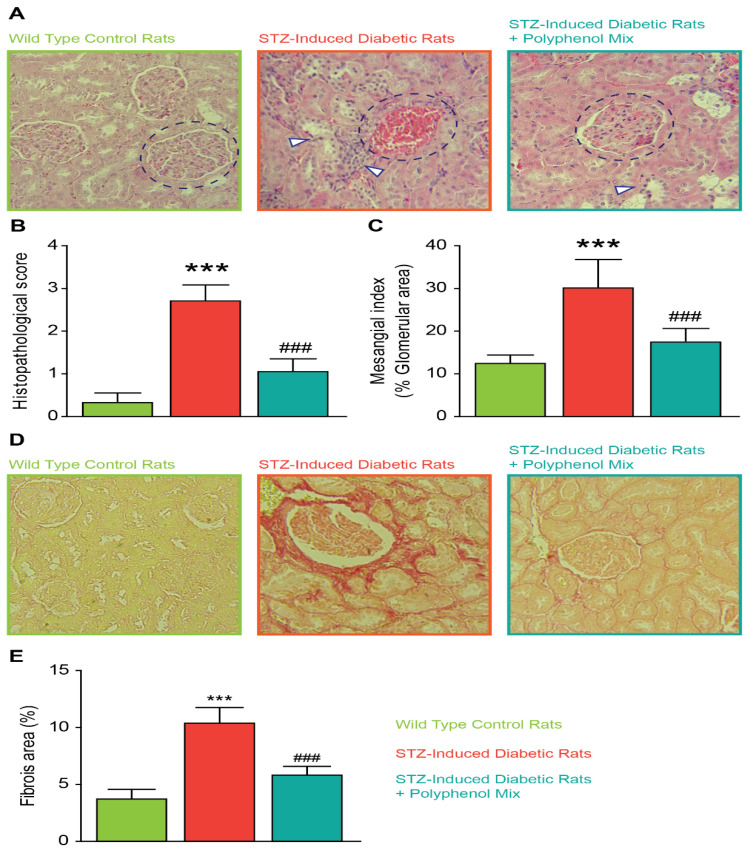
Renal histopathology and quantification. (**A**) Kidney architecture in STZ-induced diabetic rats. Histological examination (H&E stained) (400×) in rat kidney of normal group, STZ-induced diabetic group, untreated or treated with the polyphenol mix NCQ. Control kidney section showing normal histological features of glomeruli and tubules (labelled in green). STZ-induced diabetic rat’s kidney showing glomerular degeneration and hypercellularity (labelled in orange). Most of the cortical tubules showed morphologic changes, some of them being tubular necrosis, degeneration of tubular epithelium with interstitial hemorrhage. STZ-induced diabetic rats treated with NCQ (labelled in bleu) showing more remarkable improvement and the glomeruli were normal while renal tubules showed mild necrosis. Bowman’s capsules are highlighted in blue circles in all condition while necrotic cells and tubular epithelium labelled with white arrows. (**B**) Graph showing total histology scores for inflammation and fibrosis in each group. (**C**) The mesangial matrix index was defined as the proportion of the glomerular tuft occupied by the mesangial matrix area (excluding nuclei). (**D**) Renal interstitial structure (picrosirius red stained) and (**E**) fibrosis area in each group. ImageJ software (ImageJ version 1.3v; NIH, Bethesda, MD) was used to calculate the percent area of each image that stained positive for Sirius Red. Data are expressed as means ± SEM. *** *p* < 0.001, when the control group is compared to STZ-induced diabetic rats group and ### *p* < 0.001 when STZ untreated rats are compared to NCQ-treated group.

The molecular and cellular changes in STZ-induced diabetic rats treated or non-treated with the NCQ phenolic mix are also visible in kidney architecture. Kidneys from all groups were analyzed by a histological method on sections stained with hematoxylin-eosin. Histological modifications in diabetic rats’ kidneys were characterized by structural alterations at the level of the cortical zone where Bowman’s capsules adhere to the glomeruli with consequent reduction of the urinary chamber spaces. Other tissue changes are present in the proximal, distal, and collecting tubes. More interestingly, we noticed the presence of necrotic cells and leukocytes infiltration as an indication of inflammatory reaction and modifications of the podocytes, which become hypertrophied, with a large nucleus and cytoplasmic lipid droplets. However, the administration of the NCQ phenolic mix partially restored the normal kidney histological and function (Figure 4A,D). We scored the histopathology in kidney tissue with and without NCQ administration in STZ-induced diabetic rats in Figure 4B. Furthermore, we found in diabetic rats significant damage in the glomeruli, especially hypertrophy, hypercellularity, tubules dilatation, and atrophy. We scored the glomerular mesangial matrix in Figure 4C. Additionally, as compared to the control rats, picrosirius red staining showed a moderate fibrosis in diabetic rats, and the quantification demonstrated a significant difference between the groups. NCQ treatment restored the fibrotic area in rat’s kidney (Figure 4D,E).

## 4. Discussion

In the present study we investigated the protective effect of a mix constituted by natural polyphenolic compounds, namely naringin, chlorogenic acid, and quercetin (NCQ) against renal pathologies in STZ-induced diabetic rats. These food polyphenols are associated with coffee consumption and combination of grape and citrus fruits [49]. This diet is typical of Mediterranean area and de facto Tunisia [50].

Our study focuses on the type 2 diabetes which is increasing worldwide for genetics and lifestyle causes, especially diet, and the tendency is accrue in North Africa [51], given all his importance to this kind of investigation, meaning finding preventive in natural solutions for serious metabolic disorders. In fact, we used STZ-induced diabetic rats as one of the most common and easy to handle in vivo model for renal failure [52].

The uptake of the NCQ polyphenolic mix equilibrated the biochemical markers in diabetic rat blood including urea nitrogen BUN, creatinine, and the uric acid levels. Moreover, we clearly demonstrated that NCQ treatment prevents the development of oxidative stress in STZ-induced diabetic rats. Altered renal biomarkers were correlated with renal failure and diabetes; that were readjusted with the mix treatment. Our biochemical results indicate that nitric oxide (NO) level, lipid peroxidation MDA, protein carbonyl PCO, and advanced oxidation protein products AOPP level remarkably decreased, while catalase activity, superoxide dismutase activity, reduced glutathione GSH level, glutathione peroxidase activity, and Na^+^-K^+^-ATPase activities significantly increased to balance the control value in comparison to diabetic rats.

Moreover, the polyphenol mix reduced the fibrotic and apoptotic sequels that were induced by the high glucose concentration. Indeed, the matrix metalloproteinase’s MMP-9 and MMP-2 and the transforming growth factor-beta TGF-β expression were assessed in these phenomena. MMP-9/MMP-2 are matrix-degrading enzymes implicated in inflammation process, among others pathways [53]. Moreover, MMP-9 has been reported to activate latent TGF-β to its active form and stimulates fibroblasts. Over expression of these proteins plays an important role in tissue remodeling and provokes fibrosis [54]. In addition, key genes of pro-inflammatory cytokines TNF-α and the transcription factor p53 involved in fibrosis and apoptosis were also highly active. Histological examination of NCQ treated rats showed a normal renal architecture correlated with the less necrotic cells, less leukocytes infiltration, and consequent inflammation besides a normal structural and functional tissue and organelles such as Bowman’s capsules or the urinary chambers. Organ dysfunction is frequently accompanied by fibrotic alterations in both diabetic people and animal models of the disease [55,56,57,58,59,60,61]. Fibrosis may have a role in the etiology of diabetic retinopathy and neuropathy as well as the pathogenesis of diabetic cardiomyopathy, nephropathy, and liver failure [55,56,57,58,59,60,61]. Long-term diabetic patients frequently have severe fibrotic lesions in their heart, kidney and liver [55,56,57,58,59,60,61]. The mechanism is unclear; however, these fibrotic alterations are caused by metabolic dysfunction, or reflect atherosclerotic disease-related consequences, or the high prevalence of other pro-fibrotic comorbid conditions, such as hypertension and dyslipidemias.

It was proved that aging stimulates inflammation response and provokes consequent renal fibrosis [62]. Indeed, during aging, inflammatory mediators are released and activate fibroblasts to initiate renal fibrosis [63,64]. Chronic inflammation and oxidative stress are the main mechanisms of age-related renal fibrosis. Interestingly, previous reports showed that antioxidant interventions such as calorie restriction or resveratrol or spermidine administration in rats increased lifespan and reduced kidney injuries [65]. This finding suggests that fibrosis induced by aging could also be delayed or reversed with natural antioxidants, including polyphenols.

Our work correlates with previous reports showing the effect of natural polyphenols uptakes in preventing metabolic diseases and especially diabetes and renal failure. Indeed, recent studies correlate the moderate uptake of polyphenols with preventive effect against chronic kidney disease [66,67,68,69]. Moreover, polyphenols consumption was also advised as a cure for patients undergoing dialysis in hospital service [70] and for those who are increasingly prescribed clinical treatment for kidney disease and renal fibrosis [69,71,72]. Despite the large heterogeneity of the chronic kidney disease patients, including the genetic and the behavior causes, food polyphenols present as a large spectrum and safe drugs complements as demonstrated with the increase of human clinical trials in the recent years [73]. However, this tendency should be better supervised and huge research effort is still needed to avoid any excess or uncalculated synergy that could occur before clinical approval, especially due to the critical pharmacokinetics and extensive metabolization that polyphenols undergo in the human body [66].

Our study needs further investigations in order to present a thorough description of the physio-pathological pathway of naringin, chlorogenic acid, and quercetin phenols mix in preventing renal failure and restoring kidney functions in vivo.

## 5. Conclusions

In conclusion, our study described for the first time the positive effect on renal and kidney functions after administration of a mix of natural polyphenols composed of naringin (50 mg/kg), chlorogenic acid (10 mg/kg), and quercetin (50 mg/kg) in STZ-induced diabetic rats. In our in vivo model, NCQ supplementation repaired renal function and reversed renal pathological changes (renal interstitial fibrosis, leukocyte infiltration and the glomerular degeneration). The effect is due to an antioxidant stress mechanism, as well as the inhibition of the transforming growth factor-beta TGF-β, tumor necrosis factor TNFα and p53. Taken together, NCQ mix could, thus, be used as a dietary supplement to prevent diabetic nephropathy. In perspective, the investigation of other biomarkers, especially in rat urine, as faster and easier methods to access kidney damage could help to investigate other polyphenols and other mix of polyphenols to prevent nephropathy.

## Figures and Tables

**Table 1:** Primers and probes used in quantitative real-time PCR Analysis.

Aim Gene	Oligonucleotide Sequence
MMP-2	(F) 5′ CGTGGTGAGATCTTCTTCTTCAAGGA 3′ (R) 5′ CCTCATACACAGCGTCAATCTTTTC 3′
MMP-9	(F) 5′AATTCGACTTGAAGTCTCAGAAGG 3′ (R) 5′ATTAGGTGACCCTGTCGCTG 3′
P53	(F) 5′ CCCCTGAAGACTGGATAACTG 3′ (R) 5′ AAGTATTTGTCATGGCAGAAATAGG 3′
TNF-α	(F) 5′AAATGGGCTCCCTCTCATCAGTTC 3′ (R) 5′ TCTGCTTGGTGGTTTGCTACGAC 3′
TGF-β	(F) 5′GGGCTTTCGCTTCAGTGCT 3′ (R) 5′TCGGTTCATGTCATGGATGGT 3′
β–actin	(F) 5′GGAGATTACTGCCCTGGCTCCTA 3′ (R) 5′GACTCATCGTACTCCTGCTTGCTG 3′

## Data Availability

Not applicable.
